# The Correlation of Paternal Age on Semen Parameters in Assisted Reproduction: A Retrospective Study in Qassim, Saudi Arabia

**DOI:** 10.7759/cureus.61632

**Published:** 2024-06-04

**Authors:** Badr Alharbi, Fuhaid Alqossayir, Adel Moalwi, Emad Alwashmi, Adel H Alharbi, Abdullah Aloraini, Arwa Aljumah, Manahil Alhomaidhi, Mohammed Almansour

**Affiliations:** 1 Department of Surgery, College of Medicine, Qassim University, Buraydah, SAU; 2 Department of Family and Community Medicine, College of Medicine, Qassim University, Buraydah, SAU; 3 Department of Surgery, College of Medicine, Najran University, Najran, SAU; 4 Department of Internal Medicine, College of Medicine, Qassim University, Buraydah, SAU; 5 Department of Reproductive Medicine, Prince Faisal Bin Mishaal Fertility Center, Buraydah, SAU; 6 Department of Urology, Imperial College London, London, GBR; 7 Department of Urology, King Fahd Specialist Hospital, Buraydah, SAU

**Keywords:** epidemiology, male fertility, assisted reproductive technology, semen quality, advanced paternal age

## Abstract

Introduction: In the past, fertility concerns have predominantly revolved around the effect of a woman's age on the quality of her eggs and the success of her pregnancy. While men generally retain their ability to father children throughout their lives, there is evidence suggesting a decline in natural conception rates as paternal age increases. A growing body of research indicates a potential link between advanced paternal age (APA) and various adverse outcomes, including changes in sperm genetics, reduced conception rates, higher rates of miscarriage, lower live birth rates, and even long-term health consequences in offspring. However, it remains unclear whether there is an association between APA and the effectiveness of assisted reproductive technology (ART). This study aims to shed light on the relationship between APA and semen parameters.

Methodology: This is a retrospective, descriptive study analyzing data from electronic medical records of men undergoing ART at a fertility clinic in Saudia Arabia (2017-2022). Men aged 21-60 with at least one semen analysis and no missing data/hormonal treatment were included. Data on age and semen parameters (count, motility, and morphology) were extracted and analyzed using Jeffreys's Amazing Statistics Program (JASP; University of Amsterdam, Amsterdam, Netherlands) (descriptive statistics, Spearman's rank correlation).

Results: Analysis of 1506 men undergoing ART revealed a mean age of 37 years (SD=6.94) and a mean sperm count of 55.0 million/mL (SD=46.05). The correlation between age and sperm count indicates a minimal association (r=0.075, p<0.01); moderate positive correlations were observed between sperm count and motility (r=0.406); count and morphology (r=0.543); and motility and morphology (r=0.458).

Conclusion: Age may not be a major factor in overall sperm parameters for this population, but a strong positive correlation was observed between sperm count, motility, and normal morphology. These findings suggest that these semen parameters are interconnected, with higher sperm counts potentially indicating better overall sperm quality.

## Introduction

Traditionally, fertility consultations have prioritized a woman's age due to its impact on egg quality, directly affecting a couple's ability to conceive naturally and achieve successful pregnancy outcomes. This emphasis stems from the well-established negative effects of advanced maternal age on fertility, particularly in assisted reproductive technologies (ARTs) [1,2]. Advanced maternal age is linked to higher rates of miscarriage, obstetric difficulties, and neonatal mortality, as well as reduced success of ART [3].

In recent years, there has been an increasing trend among couples to delay having children until they are older. This is also reflected in the rising average age of fathers when they have their first child in developed nations across the globe [4,5]. The level of understanding regarding the influence of older paternal age on the health of offspring in the general population of Saudi Arabia is very limited [6]. Despite the substantial amount of research and evidence pertaining to the relationship between maternal age and reproductive outcomes, there is still an ongoing debate regarding the data on how paternal age affects fertility.

Research suggests that the chances of natural conception decrease as a man's age increases, even though his ability to father a child typically persists throughout his life [7,8]. Studies have reported a potential association between advanced paternal age (APA) and a range of negative outcomes, including altered sperm genetics, decreased conception rates, increased miscarriage rates, lower live birth rates, and even long-term health consequences in offspring [9,10]. Furthermore, babies born to older fathers are more likely to experience negative delivery outcomes such as convulsions in newborns, low birth weight, and the need for admission to the neonatal critical care unit [11]. Research suggests a correlation between APA and the occurrence of various psychiatric and neurocognitive disorders in offspring [12]. These disorders include schizophrenia, autism spectrum disorder (ASD), and obsessive-compulsive disorder (OCD). Additionally, APA has been linked to an increased risk of childhood cancers [10].

Research has indicated that APA is linked to a reduction in semen volume [13]. Furthermore, studies have shown that there is a clear decrease in sperm count and sperm motility among men as they age. This decline becomes significant around the ages of 41 and 41.5 [14,15].

There is a lack of consensus regarding the correlation between paternal age and the outcomes of ART. Several studies have identified potential links between APA and unfavorable outcomes in infertility treatment. These associations are believed to be due to an increased occurrence of errors in spermatogenesis in older testes, which leads to lower DNA quality and higher levels of reactive oxygen species in semen. As a result, this can lead to DNA damage and potentially hinder the activation of the male genome, thereby potentially increasing the risk of miscarriage [16]. These findings have important implications for clinical practice, highlighting the need for a comprehensive approach to male fertility evaluation and counseling, beyond sole consideration of paternal age.

However, it has also been reported that there is no link between male aging and pregnancy outcomes [17]. A study carried out at King Abdul-Aziz Medical City (KAMC) in Riyadh, Saudi Arabia discovered that the age of the father does not affect the quality of semen, factors associated with the in vitro fertilization (IVF) process, or the likelihood of pregnancy in a Saudi Arabian population [18].

The aim of this study is to evaluate the impact of APA on semen parameters in couples who underwent ART at the Prince Faisal Bin Mishaal Fertility facility in Buraydah, Qassim Region, Saudi Arabia.

## Materials and methods

Study design

This was a retrospective, descriptive study analyzing data from electronic medical records.

Setting and participants

The study was conducted at Prince Faisal Bin Mishaal Fertility Center in Buraydah, Qassim Region, Saudi Arabia. This center is the only one in the region and offers free infertility treatment. Additionally, patients from all over the country are referred to this center. The study population included all men with infertility issues and who underwent ART treatment at the center between January 2017 and December 2022. Moreover, since 2017, data have been stored in electronic medical records, thereby facilitating the analysis of such data. For this research study, the inclusion criteria consist of males who are between 21 and 60 years old and have at least one accessible semen analysis record in their medical records. The exclusion criteria include individuals who do not have essential data regarding their age or semen analysis results, as well as those who are currently undergoing hormonal treatment.

Ethical approval

This hospital-based research was obtained from the Qassim Region Research Ethics Committee, which provided the ethical approval. All procedures adhered to ethical principles and ensured participant confidentiality. The confidentiality of participants and data was ensured through various measures. Firstly, all participant data was de-identified and anonymized to safeguard confidentiality. In addition, authorized personnel were solely responsible for accessing and extracting electronic medical records. Furthermore, data was securely stored on password-protected computers and servers, with restricted access limited to research team members who had signed confidentiality agreements. Importantly, participants' names and identifying information were excluded from data collection and analysis. Lastly, data was presented in aggregate form to prevent any possibility of individual identification.

Data collection

Data were extracted from the center's electronic database of all males who visited during the study period. The following information was retrieved for each male participant: age and semen analysis results (sperm count, motility, morphology).

Data management and statistical analysis

Microsoft Excel (Microsoft® Corp., Redmond, WA, USA) was used for data cleaning and coding. Descriptive statistics were performed using Jeffreys's Amazing Statistics Program (JASP; version 0.18.1, University of Amsterdam, Amsterdam, Netherlands). Spearman's rank correlation coefficient was used to assess the relationships between age, sperm count, motility, and morphology. A p-value of <0.05 was considered statistically significant.

## Results

In this study, 1506 participants were included. Table 1 provides descriptive statistics for males assessed for infertility, focusing on age and BMI.

**Table 1:** Descriptive statistics of males assessed for infertility BMI: body mass index

Variable	Mean	Std. Deviation
Age	37.109	6.940
BMI	29.947	6.636

Table 2 shows detailed descriptive statistics for various parameters of males' sperm assessed for infertility. There are 1363 valid entries for sperm count (MIL/mL), 1455 for semen volume, 1346 for sperm progressive motility, and 1323 for the percentage of sperm with normal morphology.

**Table 2:** Descriptive statistics of different parameters of male sperms assessed for infertility

Variables	Valid	Missing	Mean	SD
Sperm count (MIL/mL)	1363	143	55.00	46.005
Semen volume (mL)	1455	51	2.531	1.606
Sperm motility	1346	160	46.596	21.267
Sperm normal morphology %	1323	183	5.135	3.925

Table 3 presents Spearman's correlation coefficients for male infertility parameters. The correlation between age and sperm count indicates a minimal association (r=0.075, p<0.01). This suggests that increasing age has little connection with sperm counts. Additionally, a negligible negative non-significant correlation (r=-0.052, p=0.054) is reported between age and motility, indicating a slight association. Similarly, age and normal sperm morphology exhibit a weak positive correlation (r=0.027, p=0.325), although not statistically significant.

**Table 3:** Correlation between different parameters of males for the assessment for infertility p<0.05, statistically significant

Variables	Variables	Spearman's rho	p-value	Lower 95% CI	Upper 95% CI
Age	Sperm count (MIL/ml)	0.075	0.006	0.022	0.127
Age	Sperm motility	-0.052	0.054	-0.106	9.736×10^-4^
Age	Sperm normal morphology	0.027	0.325	-0.027	0.081
Sperm count (MIL/mL)	Sperm motility	0.406	<0.001	0.360	0.450
Sperm count (MIL/mL)	Sperm normal morphology	0.543	<0.001	0.504	0.580
Sperm motility	Sperm normal morphology	0.458	<0.001	0.414	0.499

On the other hand, there are strong positive correlations between sperm count and motility (r=0.406, p<0.001), sperm count and normal morphology (r=0.543, p<0.001), and motility and abnormal morphology (0.458, p<0.001). The significant moderate positive correlation (r=0.406) between sperm counts and motility indicates that as the sperm count increases, the motility tends to increase as well. Moreover, there is a substantial association (r=0.543) between sperm count and the percentage of normal morphology. Additionally, there is a significant correlation (r=0.458) between motility and normal morphology.

## Discussion

This study sought to investigate the correlation between APA and semen parameters. The study found no correlation between parental age, sperm count, or sperm motility. Moreover, a moderately positive correlation has been observed between sperm count and normal sperm motility and morphology.

The impact of paternal age on reproductive outcomes has received considerable attention in recent years. APA, generally defined as fatherhood above the age of 40 [19], is becoming increasingly common in contemporary society. Our study, which included a substantial cohort of 1506 patients, provided a detailed view of the demographics and semen parameters. We observed that the mean age of the male patients was approximately 37 years, reflecting the trend of delayed fatherhood [20]. The semen parameters provided the baseline data set, laying the groundwork for our examination of the influence of paternal age on male fertility [21]. To elucidate the relationship between male age and semen parameters, we conducted Spearman's correlation analysis. Importantly, we found a positive correlation between male age and sperm count. This suggests that as paternal age increases, there is a slight increase in sperm count, contrary to some previous studies that found a decrease in sperm count with increasing paternal age [13,16]. APA has been associated with decreased testosterone levels, which can influence semen quality and quantity. Older men may undergo changes in the hypothalamic-pituitary-testicular axis, impacting sperm production and maturation. Moreover, oxidative stress and DNA damage can accumulate with age, potentially affecting sperm quality and function. These biological mechanisms play a crucial role in understanding the intricate relationships between male age and semen parameters, with significant implications for male fertility and reproductive health [22]. However, this correlation, while statistically significant, may not have substantial clinical relevance due to the relatively low correlation coefficient. One possible explanation for the positive correlation between paternal age and sperm concentration is that as men age, there may be a decrease in semen volume, which could lead to a perceived increase in sperm concentration.

On the other hand, the correlation between male age and sperm motility, as well as the percentage of sperm with abnormal morphology, was not significant in our study, which aligns with the study conducted by Kumar et al. (2017) [23]. This indicates that APA may have a limited impact on these specific semen parameters within our study population. Additionally, the analysis revealed positive correlations among the semen parameters themselves. Sperm count (MIL/mL) demonstrated a positive correlation with sperm motility and the percentage of sperm with normal morphology. These findings highlight the interconnected nature of these variables, underscoring the importance of considering multiple semen parameters when evaluating male fertility [24].

The impact of APA on semen parameters can vary across different populations and research settings. For example, Milardi et al. (2012) reported a more pronounced correlation between APA and semen quality [24], while Dain et al. (2011) found limited or no significant effect [25]. These discrepancies may arise from differences in sample size, demographics, and methodologies employed in these studies. Therefore, it is crucial to recognize the need for additional research to elucidate the complex relationship between APA and semen parameters, taking into account potential confounding factors. In further support of our findings, a retrospective study by Morris et al. (2021) found a positive correlation between APA and sperm count [26]. Other studies have emphasized the importance of considering other influencing factors, such as lifestyle and genetics, which can interact with APA and impact semen quality [27].

Our study has some limitations. Firstly, semen analysis was based on a single sample, which may not provide a complete picture of sperm quality. Secondly, there was a lack of data on lifestyle factors such as smoking, drinking, and medical history. Additionally, hormone levels and abstinence time were not taken into consideration. Furthermore, the study population represents a specific demographic, and the impact of age on semen parameters may vary among different ethnicities and geographic regions. Future research that includes these variables is essential for a more comprehensive understanding of the relationship between paternal age and sperm quality.

## Conclusions

There is a weak correlation between the age of males and their sperm count. Additionally, we observed a moderate positive relationship between sperm count, motility, and normal morphology. These findings suggest that APA may only have a moderate impact on these specific semen parameters. Our research highlights the importance of considering various semen characteristics when assessing male fertility. Although our study offers valuable insights, it is crucial to interpret these findings in the context of other potential factors that may influence couples' fertility journey. To gain a deeper understanding of the intricate relationship between APA and male fertility, future research should address the limitations of this study. This could involve expanding the sample to include more diverse populations and incorporating a broader range of relevant variables in the analysis.
